# Glycogen Synthase Kinase 3β Is a Key Regulator in the Inhibitory Effects of Accumbal Cocaine- and Amphetamine-Regulated Transcript Peptide 55–102 on Amphetamine-Induced Locomotor Activity

**DOI:** 10.3390/ijms232415633

**Published:** 2022-12-09

**Authors:** Bo Ram Cho, Wha Young Kim, Ju Kyong Jang, Jung Won Lee, Jeong-Hoon Kim

**Affiliations:** 1 Department of Psychiatry, Yale University School of Medicine, New Haven, CT 06519, USA; 2 Department of Physiology, Yonsei University College of Medicine, Seoul 03722, Republic of Korea; 3 Department of Pharmacology, Bio-Pharm Solutions Co., Ltd., Suwon-si 16229, Gyeonggi-do, Republic of Korea; 4 Division of In Vitro Diagnostic Devices, National Institute of Food and Drug Safety Evaluation, Cheongju-si 28159, Chungcheongbuk-do, Republic of Korea; 5 Department of Medical Sciences, Yonsei University College of Medicine, Seoul 03722, Republic of Korea

**Keywords:** amphetamine, nucleus accumbens, CART, GSK3β, locomotor activity

## Abstract

Microinjection of cocaine- and amphetamine-regulated transcript (CART) peptide 55–102 into the nucleus accumbens (NAcc) core significantly attenuates psychostimulant-induced locomotor activity. However, the molecular mechanism remains poorly understood. We examined the phosphorylation levels of Akt, glycogen synthase kinase 3β (GSK3β), and glutamate receptor 1 (GluA1) in NAcc core tissues obtained 60 min after microinjection of CART peptide 55–102 into this site, followed by systemic injection of amphetamine (AMPH). Phosphorylation levels of Akt at Thr308 and GSK3β at Ser9 were decreased, while those of GluA1 at Ser845 were increased, by AMPH treatment. These effects returned to basal levels following treatment with CART peptide 55–102. Furthermore, the negative regulatory effects of the CART peptide on AMPH-induced changes in phosphorylation levels and locomotor activity were all abolished by pretreatment with the S9 peptide, an artificially synthesized indirect GSK3β activator. These results suggest that the CART peptide 55–102 in the NAcc core plays a negative regulatory role in AMPH-induced locomotor activity by normalizing the changes in phosphorylation levels of Akt-GSK3β, and subsequently GluA1 modified by AMPH at this site. The present findings are the first to reveal GSK3β as a key regulator of the inhibitory role of the CART peptide in psychomotor stimulant-induced locomotor activity.

## 1. Introduction

The cocaine- and amphetamine-regulated transcript (CART) peptide is a neuropeptide that is abundantly expressed in the brain, including the nucleus accumbens (NAcc) [1,2,3], which mediates locomotor activation and the rewarding effects of drugs of abuse [4,5,6]. Endogenous CART peptide is post-transcriptionally processed, creating fragments of different sizes [7,8], of which the fragment comprising amino acids 55 through 102 (CART peptide 55–102) is widely used as a biologically active form [9,10]. Previous reports have found that the CART peptide 55–102 in the NAcc interacts with psychomotor stimulants to regulate their behavioral effects. For instance, microinjection of CART peptide 55–102 into the NAcc core significantly attenuated the locomotor effects of acute amphetamine (AMPH) [11] and cocaine [12], while CART peptide alone at this site did not affect locomotor activity. Furthermore, CART peptide 55–102 at this site was shown to inhibit both sensitized and conditioned locomotor activities produced by chronic psychomotor stimulants [13,14,15,16]. These results suggest that CART peptide 55–102 in the NAcc plays a negative regulatory role in psychomotor stimulant-induced locomotor activity.

A few studies have examined the signaling pathways in the NAcc that mediate the inhibitory effects of the CART peptide on psychomotor stimulants. For example, it has been shown that the blockade of cocaine-induced locomotor sensitization by the CART peptide is mediated by the inhibition of increased phosphorylation levels of extracellular signal-regulated kinase (ERK) [14] or Ca^2+^/calmodulin-dependent protein kinase II (CaMKII) [17] in the NAcc. Very recently, a CART peptide receptor, which has been thought to exist but had not been identified for a long time, was found to be an orphan G-protein-coupled receptor (GPCR) [18]. However, its downstream signals are not completely understood, except that it may be coupled with an inhibitory G-protein [19,20,21]. Regardless of its receptor identification, there is evidence that the CART peptide is generally associated with dopamine (DA)-mediated neurotransmission. For example, it has been shown that CART mRNA and peptide expression in the NAcc is regulated by DA receptors and their downstream signals, such as adenyl cyclase, protein kinase A (PKA), and cAMP response binding protein (CREB) [22,23,24].

In addition to a classic cAMP-mediated pathway, DA is known to have a distinct signaling pathway involving glycogen synthase kinase 3β (GSK3β), which reportedly contributes to DA and psychomotor stimulant-induced behaviors [25,26,27]. For instance, phosphorylation levels of GSK3β at serine 9 in the NAcc decreased following acute cocaine administration [28]. Furthermore, inhibition of GSK3β activity in the NAcc attenuated [29]—while its activation at this site enhanced [30,31]—the increase in locomotor activity induced by acute cocaine or AMPH administration. Whether GSK3β is also involved in the negative regulatory effect of the CART peptide on psychomotor stimulant-induced behavior remains unknown.

The NAcc receives glutamatergic signaling that interacts with DA, thereby contributing to addiction. For example, activation of AMPA receptors in the NAcc is necessary for drug seeking [32]. Moreover, microinjection of AMPA or NMDA receptor agonists into the NAcc augmented the behavioral sensitization response to cocaine [33,34], while the blockade of AMPA receptors prevented cocaine sensitization at this site [34,35]. Whether GSK3β interacts with AMPA receptors to regulate its phosphorylation or expression levels is not completely understood. However, it was found that GSKβ regulates the trafficking of AMPA receptors either by direct phosphorylation of AMPA receptors or by indirect phosphorylation of other molecules (e.g., PSD-95) [36,37,38,39].

Considering these factors together, in the present study, we attempted to examine whether accumbal GSK3β contributes to the negative regulatory effect of CART peptide on AMPH-induced locomotor activity, and further, whether there is any interaction with GluA1 in this process.

## 2. Results

### 2.1. CART Peptide 55–102 in the NAcc Core Blocks the AMPH-Induced Hyperlocomotor Activity

**Figure 1:**
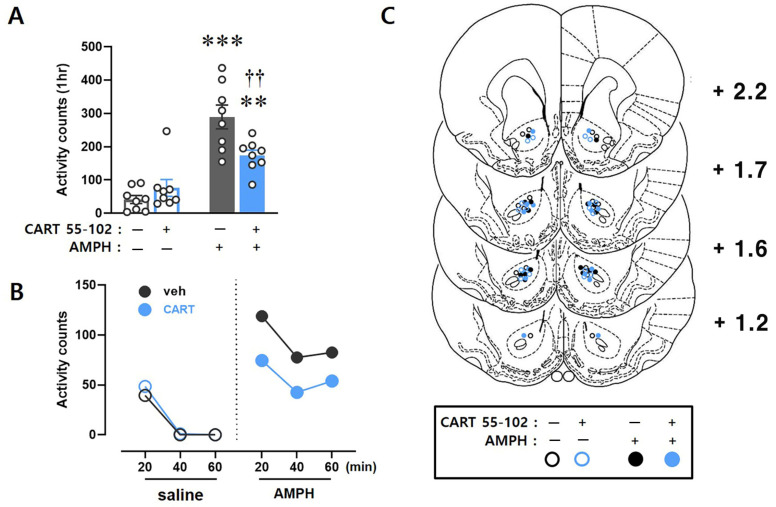
The increased locomotor activity induced by AMPH is blocked by the microinjection of CART peptide 55–102 into the NAcc core. (**A**) Data are shown as group mean (+S.E.M.) total locomotor activity counts observed during the 1 h test after bilateral microinjection of saline or CART peptide 55–102 (2.5 µg/0.5 µL/side) into the NAcc core immediately followed by saline or AMPH (1 mg/kg) IP injection. Symbols indicate significant differences revealed by post hoc Tukey comparisons following two-way ANOVA. ** *p* < 0.01, *** *p* < 0.001, significantly more counts in AMPH compared to saline IP injection. †† *p* < 0.01, significant differences between AMPH IP injections. The number of rats in each group is 8. (**B**) Time-course data are shown as group mean locomotor activity counts at 20 min intervals obtained during the 1 h following microinjection (saline or CART 55–102) + IP (saline or AMPH) injection. (**C**) Location of the injection cannula tips. All rats included in the data analyses had injection cannula tips located bilaterally in the NAcc core. The line drawings are adapted with permission from Paxinos and Watson [40]. Numbers to the right indicate millimeters from bregma.

Locomotor activity was measured for 1 h after bilateral microinjection of the CART peptide 55–102 into the NAcc core, followed by systemic AMPH administration (Figure 1). Two-way ANOVA performed on the 1 h total locomotor activity counts showed significant effects of IP injection [F_(1,28)_ = 51.261, *p* < 0.001] and microinjection x IP interaction [F_(1,28)_ = 9.838, *p* < 0.01].

Consistent with previous studies [11,16], microinjection of CART peptide 55–102 (2.5 µg/0.5 µL/side) into the NAcc core significantly inhibited the AMPH-induced hyperlocomotor activity (*p* < 0.01 by post hoc Tukey comparisons), while the basal locomotor activity was not changed by CART peptide 55–102 alone in the absence of AMPH. Time-course analyses of these findings showed that the ability of the CART peptide 55–102 to inhibit AMPH-induced hyperlocomotor activity was apparent throughout the 1 h time course (Figure 1B). The locations of the injection cannula tips used in these experiments are shown in Figure 1C.

### 2.2. CART Peptide 55–102 in the NAcc Core Inhibits the AMPH-Induced Decreases in pAkt (T308) and pGSK3β

**Figure 2:**
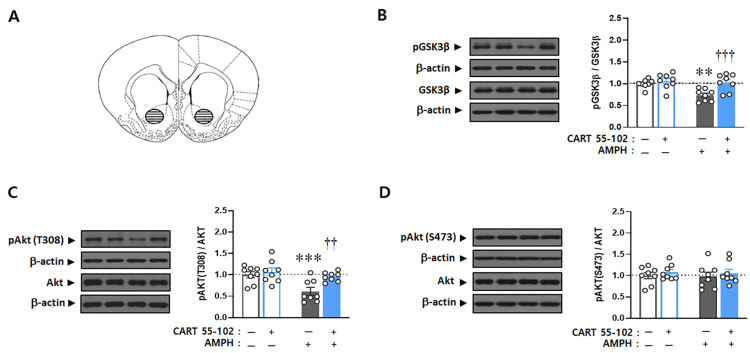
Microinjection of CART peptide 55–102 into the NAcc core inhibits the AMPH-induced decreases in phosphorylation levels of Akt (T308) and GSK3β. (**A**) The NAcc core region where tissues were sampled is shown (cross-hatched circles). Punches (1.2 mm diameter) were prepared bilaterally and pooled for each animal’s protein isolation. The line drawing is from Paxinos and Watson [40] and depicts the caudal surface of a coronal section (1.0 mm thick) extending 1.70–2.70 mm from bregma. (**B**–**D**) Representative Western blots of total and phosphorylated forms of GSK3β and Akt are shown. Values for the band intensities were first normalized to β-actin, and the average values for the ratio of phosphorylated to total proteins in each group are expressed as mean (+S.E.M.) relative to saline microinjection + saline IP injection group. Symbols indicate significant differences, as revealed by post hoc Tukey comparisons following two-way ANOVA. ** *p* < 0.01, *** *p* < 0.001, significant differences between saline microinjection. †† *p* < 0.01, ††† *p* < 0.001, significant differences within AMPH IP injection. The line drawings are adapted with permission from Paxinos and Watson [40]. The number of rats in each group is 8.

To examine whether the effect of CART peptide 55–102 on the blockade of AMPH-induced hyperlocomotor activity is under the regulation of Akt-GSK3β signaling pathway, the ratios of phosphorylated to total protein levels were studied by Western blot analysis with the NAcc core tissues (Figure 2A) obtained from rats after 1 h locomotor activity measurement. First, the phosphorylation level of GSK3β at serine 9 was examined. Two-way ANOVA performed on these data revealed multiple significant effects of microinjection [F_(1,28)_ = 10.085, *p* < 0.01], IP injection [F_(1,28)_ = 7.064, *p* < 0.05], and microinjection x IP injection [F_(1,28)_ = 4.417, *p* < 0.05]. Consistent with previous studies [25,31], acute AMPH treatment decreased the phosphorylation levels of GSK3β in the NAcc core (*p* < 0.01, by Tukey). Interestingly, this decrease in phosphorylation was inhibited, resulting in the full recovery of its phosphorylation back to the saline control level (*p* < 0.001), when CART peptide 55–102 was microinjected in the NAcc core, followed by systemic AMPH injection. CART peptide 55–102 alone did not affect GSK3β phosphorylation levels (Figure 2B).

Two-way ANOVA conducted on the phosphorylation levels of Akt at threonine 308 (T308) showed significant effects of microinjection [F_(1,28)_ = 7.248, *p* < 0.05] and IP injection [F_(1,28)_ = 10.654, *p* < 0.01]. Similar to pGSK3β, the phosphorylation of Akt at T308 in the NAcc core was decreased by AMPH (*p* < 0.001). Interestingly, microinjection of CART peptide 55–102 into the NAcc core, followed by systemic AMPH injection, normalized its phosphorylation back to the saline control level (*p* < 0.01), while CART peptide 55–102 itself did not affect its phosphorylation (Figure 2C). No significant changes were observed on another Akt phosphorylation site, serine 473 (S473), by any of these factors (Figure 2D). These data indicate that CART peptide produced its inhibitory action on AMPH-induced locomotor activity by interrupting the AMPH-induced changes of phosphorylation levels of Akt (T308) and GSK3β (Ser9).

### 2.3. CART Peptide 55–102 in the NAcc Core Inhibits the AMPH-Induced Increases in pGluA1 (S845) but Not pGluA1 (S831)

**Figure 3:**
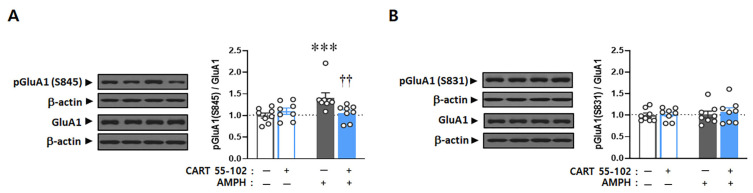
Microinjection of CART peptide 55–102 into the NAcc core inhibits the AMPH-induced increase of GluA1 phosphorylation levels at serine 845 but not at serine 831. (**A**,**B**) Representative Western blots of total and two phosphorylated forms (S845 and S831) of GluA1 are shown. Values for the band intensities were first normalized to β-actin, and the average values for the ratio of phosphorylated to total proteins in each group are expressed as mean (+S.E.M.) relative to saline microinjection + saline IP injected group. Symbols indicate significant differences, as revealed by post hoc Tukey comparisons following two-way ANOVA. *** *p* < 0.001, significant differences between saline microinjection. †† *p* < 0.01, significant differences within AMPH IP injection. The number of rats in each group is 8.

To further examine whether CART peptide 55–102 may regulate the phosphorylation levels of GluA1, the phosphorylated to total GluA1 ratio was examined by Western blot analysis with the same NAcc core tissues (Figure 2A) used for the analysis of pGSK3β and pAkt. Since GluA1 has two major phosphorylation sites, serine 845 (S845) and serine 831 (S831), changes in phosphorylation levels at both sites were simultaneously examined. Two-way ANOVA conducted on these data showed significant effects of IP injection [F_(1,28)_ = 5.283, *p* < 0.05] and microinjection x IP injection [F_(1,28)_ = 8.715, *p* < 0.01] for S845 but no significance for S831. Post hoc Tukey comparisons revealed that the AMPH-induced increase in GluA1 phosphorylation at Ser845 (*p* < 0.001) was completely blocked by microinjection of the CART peptide 55–102 into the NAcc core (*p* < 0.01) (Figure 3A). Phosphorylation levels of the Ser831 residue were not changed by AMPH, CART peptide 55–102 alone, or a combination of these two factors (Figure 3B).

### 2.4. Interruption to the Akt-GSK3β Signaling in the NAcc Core Abolishes Inhibitory Effects of CART Peptide 55–102 on AMPH

**Figure 4:**
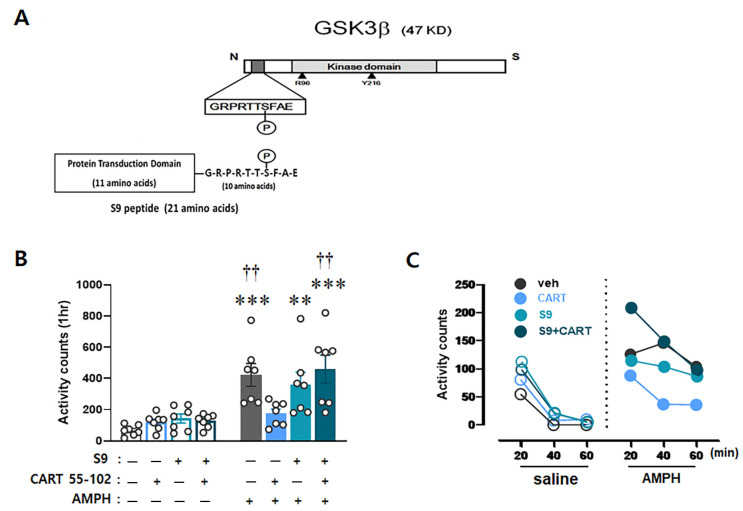
Interruption of Akt-GSK3β signaling in the NAcc core abolishes inhibitory effects of CART peptide 55–102 on AMPH-induced locomotor activity. (**A**) A diagram showing the S9 peptide with short amino acid sequences competitive against a phosphorylation site at serine 9 of endogenous GSK3β. (**B**) Data are shown as group mean (+S.E.M.) total locomotor activity counts observed during the 1 h test after the bilateral microinjection of saline, CART peptide 55–102 (2.5 µg/0.5 µL/side), or S9 peptide (5.0 µg/0.5 µL/side) into the NAcc core immediately followed by saline or AMPH (1 mg/kg) IP injection. Symbols indicate significant differences revealed by post hoc Tukey comparisons following two-way ANOVA. ** *p* < 0.01, *** *p* < 0.001, significantly more counts compared to saline—saline microinjections + saline IP injection. †† *p* < 0.01, significant differences compared to saline—CART 55–102 microinjections + AMPH IP injection. The number of rats in each group is 7. (**C**) Time-course data are shown as group mean locomotor activity counts at 20 min intervals obtained during the 1 h following microinjections (saline, CART 55–102 or S9) + IP (saline or AMPH) injection.

To verify whether AMPH-induced upregulation of GSK3β activity (i.e., decrease in GSK3β phosphorylation) contributes to increased GluA1 phosphorylation and eventually locomotor activity, the S9 peptide, which is a small synthetic peptide acting as a competitive inhibitor against the phosphorylation site of endogenous GSK3β [30] (Figure 4A), was co-microinjected with CART peptide 55–102 into the NAcc core, immediately followed by AMPH IP injection. Two-way ANOVA conducted on locomotor activity counts revealed significant effects of IP injection [F_(1,48)_ = 41.338, *p* < 0.001] and microinjection x IP injection [F_(3,48)_ = 3.414, *p* < 0.05]. Consistently, CART peptide 55–102 in the NAcc core disrupted AMPH-induced hyperlocomotor activity (*p* < 0.01). However, co-microinjection of the S9 peptide followed by CART peptide 55–102 into this site abolished the blockade effects of CART peptide 55–102 on AMPH-induced locomotor activity (*p* < 0.01) (Figure 4B). Time-course analyses of these findings showed that the ability of the S9 peptide to abolish the effects of CART peptide 55–102 on AMPH-induced hyperlocomotor activity was apparent throughout the 1 h time course (Figure 4C).

**Figure 5:**
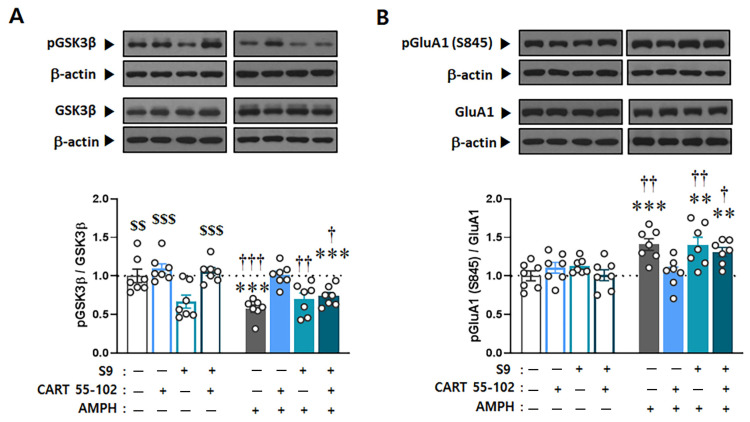
Interruption of Akt-GSK3β signaling in the NAcc core abolishes inhibitory effects of CART peptide 55–102 on AMPH-induced phosphorylation changes of GSK3β and GluA1. Representative Western blots of total and phosphorylated forms of GSK3β (**A**) and GluA1 (**B**) are shown. Values for the band intensities were first normalized to β-actin, and the average values for the ratio of phosphorylated to total proteins in each group are expressed as mean (+S.E.M.) relative to saline—saline microinjections + saline IP injection group. Symbols indicate significant differences as revealed by post hoc Tukey comparisons following two-way ANOVA. $$ *p* < 0.01, $$$ *p* < 0.001, significant differences compared to S9–saline microinjections + saline IP injection. ** *p* < 0.01, *** *p* < 0.001, significant differences compared to saline—saline microinjections + saline IP injection. † *p* < 0.05, †† *p* < 0.01, ††† *p* < 0.001, significant differences compared to saline— CART 55–102 microinjections + AMPH IP injection. The number of rats in each group is 7.

To confirm whether the S9 peptide in the Nacc core interferes with the phosphorylation levels of GSK3β and further regulates GluA1 phosphorylation, the ratio of phosphorylated to total levels of GSK3β and GluA1(S845) was evaluated. Two-way ANOVA performed on these data revealed multiple significant effects of microinjection [F_(3,48)_ = 11.396, *p* < 0.001], IP injection [F_(1,48)_ = 17.899, *p* < 0.001], and microinjection x IP injection [F_(3,48)_ = 5.097, *p* < 0.01] for GSK3β, as well as IP injection [F_(1,48)_ = 20.968, *p* < 0.001] and microinjection x IP injection [F_(3,48)_ = 4.134, *p* < 0.05] for pGluA1(S845). Similar to previous reports [30], the S9 peptide alone decreased the ratio of phosphorylated to total GSK3β levels in the NAcc core (*p* < 0.001), reconfirming that the artificially synthesized S9 peptide competes for the phosphorylation site with endogenous GSK3β (Figure 5A). CART peptide 55–102 in the NAcc core blocked the decrease in pGSK3β (*p* < 0.001) and the concurrent increase in pGluA1(S845) (*p* < 0.01) induced by AMPH. Interestingly, however, these effects were completely abolished when the S9 peptide was co-microinjected with the CART peptide 55–102, followed by AMPH IP injection (*p* < 0.01) (Figure 5A,B).

## 3. Discussion

It has been previously shown that acute AMPH or cocaine decreases the phosphorylation levels of Akt at threonine 308 residue [41,42] and GSK3β at serine 9 residue [28,30,41], resulting in the inactivation of Akt and subsequent GSK3β activation. These responses are evidently shown by Western blots in the brain areas, such as the NAcc or striatum, mediating psychomotor stimulants behaviors. Consistent with these findings, the present results show that acute AMPH administration decreased the phosphorylation levels of Akt (Thr308) and GSK3β (Ser9) in the NAcc core. Interestingly, these decreases were completely recovered to the basal level by microinjection of CART peptide 55–102 into this site (Figure 2), suggesting that it has a negative regulatory role in GSK3β signaling activated by AMPH. It is unclear whether this effect of CART peptide is mediated by nonclassical cAMP-independent DA signaling inhibition [43] or distinct phosphatidylinositol 3 (PI3) kinase-Akt-GSK3β signaling activation [44]. However, evidence suggests that PI3 kinase signaling affects Akt phosphorylation at both Thr308 and Ser473 [44], while the DA D2 receptor dephosphorylates only Thr308 [41], which is consistent with the present findings (Figure 2C,D). Further, as the CART peptide receptor is very recently identified [18] and suggestively coupled with an inhibitory G-protein [19,20,21], it is more plausible to guess that activation of the CART peptide receptor may bring secondary G-proteins, and thereby indirectly or partially release DA D2 receptor from its preformed complex with β-arrestin induced by AMPH [43]. The DA D2 receptor complex with β-arrestin decreases Akt phosphorylation by bringing protein phosphatase 2A (PP2A) close to Akt [25,41,42,43]. If the CART peptide could disrupt this process, it is possible to see the recovery of decreased phosphorylation levels of Akt, followed by GSK3β, as shown in our present results. This remains to be explored in future experiments.

There is abundant literature supporting the importance of glutamate and its interaction with DA in the NAcc mediating psychomotor stimulant-induced locomotor activity and reward [32,33,35]. For example, the administration of a DA receptor agonist into the NAcc increases extracellular glutamate and locomotor activity [45]. In contrast, pretreatment with a DA receptor antagonist blocks the stimulation of glutamate levels in the same region [46,47]. These results indicate that DA receptors simultaneously activate glutamatergic transmission. It has also been shown that acute AMPH increases the phosphorylation levels of GluA1 at Ser845, but not at Ser831, between 15 min and 60 min in the striatum and forebrain [48,49]. As the CART peptide 55–102 regulates AMPH-activated DA signaling in the NAcc, it will be interesting to elucidate whether it is also involved in glutamatergic transmission. Consistent with previous findings [48,49], we found that acute AMPH significantly increased the phosphorylation levels of GluA1 at Ser845, but not at Ser831, when examined at 60 min (Figure 3). As the phosphorylation of GluA1 at Ser845 more likely sends AMPA receptors toward synaptic site [50], it may thereby contribute to produce locomotor activity. Interestingly, however, this increase in Ser845 phosphorylation induced by AMPH was completely blocked by microinjection of the CART peptide 55–102 into the NAcc core (Figure 3A). These results demonstrate that the accumbal CART peptide regulates glutamatergic transmission as well as DA, contributing to the inhibition of AMPH-induced locomotor activity.

It has been shown that GSK3β is closely bound to GluA1 to regulate its activity in the hippocampus [36], and the GSK3β inhibitor decreases the surface and total protein levels of GluA1 in the prefrontal neurons [37]. In the same line with these findings, our present results suggest that phosphorylation of GluA1 is possibly under the regulation of GSK3β in the NAcc core, and CART peptide 55–102 disrupts this signal pathway by increasing GSK3β phosphorylation. However, as it is known that other kinases also influence phosphorylation of GluA1 at Ser845 (e.g., protein kinase A) [50], it is not certain whether the increase in its phosphorylation by AMPH is directly caused by activation of GSK3β or indirectly through other kinases. To examine whether GluA1 is under the regulation of GSK3β and CART peptide 55–102 disrupts this signal pathway, we co-microinjected S9 peptide, a potential activator for GSK3β, by competing with Akt against its phosphorylation, [30,51,52], with CART peptide 55–102 and measured phosphorylation levels of GSK3β and GluA1. As shown in Figure 5, the blockade effect of CART peptide 55–102 on AMPH-induced changes in GSK3β and GluA1 signaling was nullified in the presence of the S9 peptide, subsequently exerting the same effects on the locomotor activity (Figure 4). These results indicate that GSK3β more likely regulates the Ser845 phosphorylation level of GluA1, and interruption of this pathway in the NAcc core is a crucial step for the CART peptide to negatively regulate AMPH-induced locomotor activity. However, the signaling pathways by which GSK3β reaches GuA1 must be explored in the future.

It is worth mentioning that there exist limitations in our study. First, we reduced phosphosylation levels of GSK3β by using S9 peptide, a competitor with Akt, against the GSK3β phosphorylation site. Although it provides valuable information about how the phosphorylation levels of GSK3β are important in the inhibitory effects of CART peptide 55–102 on AMPH-induced locomotor activity, we may also need to show whether similar results come out by using a direct Akt inhibitor. Second, we showed that the phosphorylation levels of GluA1 are under the GSK3β activation. Although we cited the literature showing that GSK3β is closely bound to GluA1 to regulate its activity in the hippocampus [36], we did not directly measure whether it is also the case in the NAcc. Third, we targeted the core of the NAcc based on our previous studies [11,16,30]. Considering that two substructures of the NAcc—core and shell—are distinguished in anatomical structures and behavioral output functions [53,54,55], it will be necessary to explore signaling pathways mediating the CART effects in the shell.

**Figure 6:**
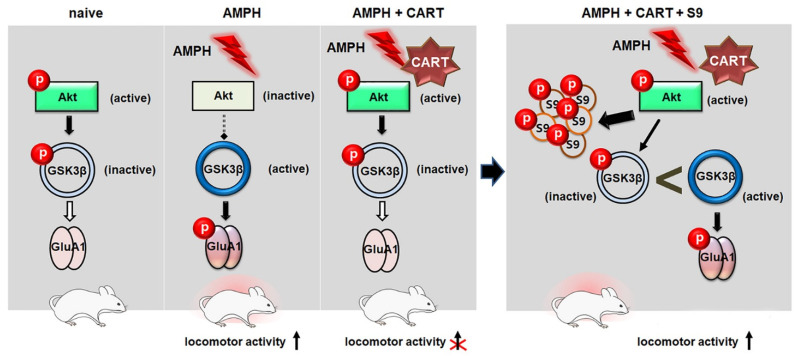
A hypothetical model incorporating present findings illustrates a signal pathway where AMPH and CART peptide 55–102 interact. The left panel shows that acute AMPH decreases phosphorylation levels of Akt and GSK3β with a concurrent increase of phosphorylation levels of GluA1 in the NAcc core, which increases locomotor activity. These effects are blocked by CART peptide 55–102 in the NAcc core. The right panel shows that the presence of S9 peptide, by competing with endogenous GSK3β against its phosphorylation site, abolishes the inhibitory action of CART peptide on the phosphorylation levels of GSK3β and GluA1, and consequently, locomotor activity induced by AMPH.

The present results showing how AMPH and the CART peptide 55–102 interact to regulate GSK3β signaling in the NAcc core are summarized in Figure 6. When AMPH is administered to rats, the phosphorylation levels of Akt and GSK3β are decreased in the NAcc core (Figure 2B,C), which inactivates Akt and subsequently activates GSK3β (second left panel, Figure 6). As one of the multiple downstream substrates, GluA1 is phosphorylated by GSK3β (Figure 3A) and contributes to increased locomotor activity (Figure 1). However, these effects are blocked when the CART peptide 55–102 is present in the NAcc core, resulting in the phosphorylation levels of all molecules (Akt, GSK3β, and GluA1) returning to basal levels (Figure 2 and Figure 3). Interestingly, in the presence of the S9 peptide, these negative effects of CART peptide 55–102 were all nullified, causing locomotor activity to increase again (right panel, Figure 6). It is worth mentioning that this schematic illustration has been simplified only based on our present findings. There may be other players affecting this signaling pathway. For example, ERK and CaMKII in the NAcc have been shown to involve in the inhibitory effects of CART peptide on psychomotor stimulant-induced locomotor activity [14,17]. As the signaling pathways of ERK and CaMKII are under the regulation of DA and glutamate, it is possible that they may interact with the CART-mediated signal pathway somewhere at the downstream effectors such as PKA or CREB [17,22,23,24,56]. How they intervene in the GSK3β pathway in terms of CART effects depicted in this study remains unexplored and needs to be more refined in the future.

## 4. Materials and Methods

### 4.1. Subjects

Male Sprague Dawley rats, 6 weeks old, weighing 200–230 g on arrival, were obtained from Orient Bio Inc. (Seongnam-si, South Korea). The rats were housed three per cage and had access to water and food ad libitum at all times. Colony rooms had a controlled room temperature (21 °C) and a 12 h light/dark cycle (lights on at 8:00 am), and all experiments were conducted during the day. All animal use procedures were conducted according to an approved Institutional Animal Care and Use Committee protocol of Yonsei University College of Medicine (Ref. No. 2012-0071).

### 4.2. Drug and Peptides

D-amphetamine sulfate (cat. no. 1180004, United States Pharmacopeial, Rockville, MD, USA) and rat CART peptide 55–102 (cat. no. 46-2-55, American Peptide, Sunnyvale, CA, USA) were dissolved in sterile 0.9% saline to a final working concentration of 1 mg/mL and 5 µg/µL, respectively. S9 peptide consists of 21 amino acids (a.a.) that include a small peptide (11 a.a.), commonly referred to as protein transduction domain [57], and a portion (10 a.a.) of the N-terminus sequence of GSK3β (GRPRTTSFAE) known as the substrate site for Akt, and thereby competes with GSK3β against its phosphorylation [51,52] (see Figure 4A). It was artificially synthesized and kindly provided by Professor Soo Young Lee at the Center for Cell Signaling and Drug Discovery Research, Ewha Womans University (Seoul, South Korea). It was dissolved in sterile 0.9% saline to a final working concentration of 10.0 μg/µL.

### 4.3. Surgery

Rats were anesthetized with intraperitoneal (IP) ketamine (100 mg/kg) and xylazine (6 mg/kg), placed in a stereotaxic instrument with the incisor bar at 5.0 mm above the interaural line, and implanted with chronic bilateral guide cannulas (22 gauge; Plastics One, Roanoke, VA, USA) aimed at the NAcc core (A/P, +3.4; L, ±1.5; D/V, –7.5 mm from bregma and skull) [58]. Cannulas were angled at 10° to the vertical, positioned 1 mm above the final injection site, and secured with dental acrylic cement anchored to stainless steel screws fixed to the skull. After surgery, 28 gauge obturators were placed in the guide cannulas, and rats were returned to their home cages for a 7-day recovery period.

### 4.4. Intracranial Microinjection

Bilateral intracranial microinjections into the NAcc core were made in the freely moving rat. Injection cannulas (28 gauge) connected to 1 µL syringes (Hamilton, Reno, NV, USA) via PE-20 tubing were inserted to a depth 1 mm below the guide cannula tips. Injections were made in a volume of 0.5 µL per side over 30 s. After 1 min, the injection cannulas were exchanged to the obturators and rats stayed in the home cage for another 1 min to give enough time for drug to diffuse in the NAcc core. Then, rats were placed in the activity boxes immediately after IP injection.

### 4.5. Locomotor Activity

Locomotor activity was measured with a bank of 9 activity boxes (35 × 25 × 40 cm) (IWOO Scientific Corporation, Seoul, Korea) made of translucent Plexiglas. Each box was individually housed in a PVC plastic sound-attenuating cubicle. The floor of each box consisted of 21 stainless steel rods (5 mm diameter) spaced 1.2 cm apart center-to-center. Two infrared light photobeams (Med Associates, St. Albans, VT, USA), positioned 4.5 cm above the floor and spaced evenly along the longitudinal axis of the box, were used to estimate horizontal locomotor activity.

### 4.6. Brain Tissue Preparation

Rats were decapitated by guillotine immediately after locomotor activity measurement. Brains were rapidly removed and coronal sections (1.0 mm thick extending 1.60–2.60 mm from bregma) were obtained with an ice-cold brain slicer (Model SA-2160, Roboz Surgical Instrument Co., Gaithersburg, MD, USA). The location for the injection cannula tips was verified and recorded. Then, the NAcc core tissues were obtained in the circular punch with 1.2 mm diameter on an ice-cold plate (see Figure 2A), immediately frozen on dry ice and stored at –80 °C. They were prepared bilaterally and pooled for each individual animal’s protein isolation.

### 4.7. Western Blot

Tissues were homogenized in lysis buffer containing 0.32 M sucrose, 2 mM EDTA, 1% SDS, 10 µg/mL aprotinin, 10 µg/mL leupeptin, 1 mM phenylmethylsulfonyl fluoride, 10 mM sodium fluoride, and 1 mM sodium orthovanadate. The concentration of protein was determined by using Pierce Coomassie Protein Assay Kit (Thermo Scientific Inc., Rockford, IL, USA). Samples were then boiled for 10 min and subjected to SDS-polyacrylamide gel electrophoresis. Proteins were separated and transferred electrophoretically to nitrocellulose membranes (Bio-Rad, Hercules, CA, USA), which were then blocked with 5% bovine serum albumin (BSA) in PBS-T buffer [10 mM phosphate-buffered saline plus 0.05% Tween-20]. Antibodies used to probe the blots were as follows: total Akt (cat. No. 9272, 1:4000), phosphor-Akt (specific to detect phosphorylated Akt at threonine 308, cat. No. 9275, 1:500, and at serine 473, cat. No. 9271, 1:2000), total GSK3β (cat. No. 9315, 1:20,000), phosphor-GSK3β (specific to detect phosphorylated GSK3β at serine 9, cat. No. 9336, 1:1000), purchased from Cell Signaling (Beverly, MA, USA) and diluted in PBS-T with 5% BSA; total GluA1 (cat. No. AB1504, 1:4000). Phospho-GluA1 (specific to detect phosphorylated GluA1 at serine 845, cat. No. AB5849, 1:1000, and at serine 831, cat. No. AB5847, 1:1000), purchased from Millipore (Billerica, MA, USA) and diluted in PBS-T with 5% BSA; β-actin (cat. No. ab6276, 1:10,000), purchased from Abcam (Cambridge, UK) and diluted in PBS-T with 5% skim milk. Two separate gels were used to detect total and phosphorylated proteins, respectively. Primary antibodies were detected with peroxidase-conjugated secondary antibodies, anti-rabbit IgG (cat. No. K0211708, 1:2000; KOMA Biotech, Seoul, Korea) or anti-mouse IgG (cat. No. 7076 1:5000; Cell Signaling), diluted in PBS-T with 5% skim milk, followed by enhanced chemiluminescence (ECL) reagents (cat. no. LF-QC0101, Abfrontier Co., Ltd., Seoul, South Korea) and exposure to X-ray film. Band intensities were quantified based on densitometric values using Fujifilm Science Lab 97 Image Gauge software (version 2.54) (Fujifilm, Tokyo, Japan).

### 4.8. Design and Procedures

Upon arrival, all rats were allowed a weeklong adaptation period in the new housing environment, and two separate experiments were conducted.

Experiment 1 (microinjection of CART peptide 55–102): Rats were randomly assigned to four groups after surgical recovery period and habituated in the locomotor activity boxes for 1 h to adjust to the new environment. Each group then received bilateral microinjections into the NAcc core either of saline or CART peptide 55–102 (2.5 µg/0.5 µL/side), immediately followed by a single IP injection of saline or AMPH (1 mg/kg). Immediately after the IP injection, rats were placed back in the locomotor activity boxes, and their locomotor activity was measured for 1 h. After an hour, they were decapitated, and their brain tissues (the NAcc core) were prepared for Western blot analysis. The dose for CART peptide 55–102 was chosen based on previous findings, indicating that it blocks AMPH-induced hyperlocomotor activity but does not affect basal locomotor activity [11,16]. A total of 32 rats were used, and all were included in the statistical analysis.

Experiment 2 (co-microinjection of S9 peptide with CART peptide 55–102): Rats were randomly assigned to eight groups after the surgical recovery period and habituated in locomotor activity boxes for 1 h to adjust to the new environment. Each group received bilateral microinjections into the NAcc core twice with a 5 min interval, first with saline or S9 peptide (5.0 µg/0.5 µL/side), second with saline or CART peptide 55–102 (2.5 µg/0.5 µL/side), immediately followed by a single IP injection of saline or AMPH (1 mg/kg). Immediately after the IP injection, rats were placed back in the locomotor activity boxes, and their locomotor activity was measured for 1 h. After an hour, they were decapitated, and their brain tissues (the NAcc core) were prepared for Western blot analysis. The dose for the S9 peptide was chosen based on previous findings that this dose did not affect basal locomotor activity, while it significantly decreased the phosphor-GSK3β levels at this site [30]. A total of 56 rats were used and all included in the statistical analysis.

### 4.9. Statistical Analysis

Statistical analyses were performed using the Sigma Plot version 12.0 (Systat Software, San Jose, CA, USA). The data were analyzed with two-way analysis of variance (ANOVA), followed by post hoc Tukey comparisons. Differences between experimental conditions were considered statistically significant when *p* < 0.05.

## 5. Conclusions

In this study, we found that the inhibitory effects of the accumbal CART peptide 55–102 on AMPH-induced locomotor activity were mediated by blockade of the AMPH-induced decrease in GSK3β phosphorylation. Furthermore, we showed that GSK3β regulates GluA1 and the accumbal CART peptide 55–102 interrupts this signal pathway. This is the first direct demonstration, to our knowledge, that GSK3β plays a pivotal role in mediating the inhibitory effects of the CART peptide 55–102 in the NAcc core on AMPH-induced locomotor activity. These findings will expand our understanding of CART peptide-regulated signal pathways in terms of their negative regulatory role in psychomotor stimulant effects.

## Data Availability

The data presented in this study are available from the corresponding author upon reasonable request.

## Abbreviations

CART: cocaine- and amphetamine-regulated transcript; NAcc: nucleus accumbens; GSK3β: glycogen synthase kinase 3β; GluA1: glutamate receptor 1; AMPH: amphetamine; ERK: extracellular signal-regulated kinase; CaMKII: Ca^2+^/calmodulin-dependent protein kinase II; GPCR: G-protein-coupled receptor; DA: dopamine; CREB: cAMP response binding protein; ANOVA: analysis of variance; IP: intraperitoneal; SEM: standard error of mean; BSA: bovine serum albumin; PBS: phosphate buffered saline; ECL: enhanced chemiluminescence.

## References

[1] Couceyro P.R., Koylu E.O., Kuhar M.J. (1997). Further studies on the anatomical distribution of CART by in situ hybridization. J. Chem. Neuroanat..

[2] Koylu E.O., Couceyro P.R., Lambert P.D., Kuhar M.J. (1998). Cocaine- and amphetamine-regulated transcript peptide immunohistochemical localization in the rat brain. J. Comp. Neurol..

[3] Ong Z.Y., McNally G.P. (2020). CART in energy balance and drug addiction: Current insight and mechanisms. Brain Res..

[4] Cador M., Bjijou Y., Stinus L. (1995). Evidence of a complete independence of the neurobiological substrates for the induction and expression of behavioral sensitization to amphetamine. Neuroscience.

[5] Robinson T.E., Berridge K.C. (1993). The neural basis of drug craving: An incentive-sensitization theory of addiction. Brain Res. Brain Res. Rev..

[6] Perugini M., Vezina P. (1994). Amphetamine administered to the ventral tegmental area sensitizes rats to the locomotor effects of nucleus accumbens amphetamine. J. Pharmacol. Exp. Ther..

[7] Koylu E.O., Couceyro P.R., Lambert P.D., Ling N.C., DeSouza E.B., Kuhar M.J. (1997). Immunohistochemical localization of novel CART peptides in rat hypothalamus, pituitary and adrenal gland. J. Neuroendocrinol..

[8] Smith Y., Koylu E.O., Couceyro P., Kuhar M.J. (1997). Ultrastructural localization of CART (cocaine- and amphetamine-regulated transcript) peptides in the nucleus accumbens of monkeys. Synapse.

[9] Thim L., Kristensen P., Nielsen P.F., Wulff B.S., Clausen J.T. (1999). Tissue-specific processing of cocaine- and amphetamine-regulated transcript peptides in the rat. Proc. Natl. Acad. Sci. USA.

[10] Dylag T., Kotlinska J., Rafalski P., Pachuta A., Silberring J. (2006). The activity of CART peptide fragments. Peptides.

[11] Kim J.H., Creekmore E., Vezina P. (2003). Microinjection of CART peptide 55-102 into the nucleus accumbens blocks amphetamine-induced locomotion. Neuropeptides.

[12] Jaworski J.N., Kozel M.A., Philpot K.B., Kuhar M.J. (2003). Intra-accumbal injection of CART (cocaine-amphetamine regulated transcript) peptide reduces cocaine-induced locomotor activity. J. Pharmacol. Exp. Ther..

[13] Jaworski J.N., Hansen S.T., Kuhar M.J., Mark G.P. (2008). Injection of CART (cocaine- and amphetamine-regulated transcript) peptide into the nucleus accumbens reduces cocaine self-administration in rats. Behav. Brain Res..

[14] Yoon H.S., Kim S., Park H.K., Kim J.H. (2007). Microinjection of CART peptide 55-102 into the nucleus accumbens blocks both the expression of behavioral sensitization and ERK phosphorylation by cocaine. Neuropharmacology.

[15] Yoon H.S., Kim W.Y., Kim J.H. (2010). Microinjection of CART peptide 55-102 into the nucleus accumbens core inhibits the expression of conditioned hyperactivity in a cocaine-associated environment. Behav. Brain Res..

[16] Kim S., Yoon H.S., Kim J.H. (2007). CART peptide 55-102 microinjected into the nucleus accumbens inhibits the expression of behavioral sensitization by amphetamine. Regul. Peptides.

[17] Xiong L., Meng Q., Sun X., Lu X., Fu Q., Peng Q., Yang J., Oh K.W., Hu Z. (2018). Cocaine- and amphetamine-regulated transcript peptide in the nucleus accumbens shell inhibits cocaine-induced locomotor sensitization to transient over-expression of -Ca^2+^/calmodulin-dependent protein kinase II. J. Neurochem..

[18] Yosten G.L.C., Harada C.M., Haddock C., Giancotti L.A., Kolar G.R., Patel R., Guo C., Chen Z., Zhang J., Doyle T.M. (2020). GPR160 de-orphanization reveals critical roles in neuropathic pain in rodents. J. Clin. Investig..

[19] Yermolaieva O., Chen J., Couceyro P.R., Hoshi T. (2001). Cocaine- and amphetamine-regulated transcript peptide modulation of voltage-gated Ca2+ signaling in hippocampal neurons. J. Neurosci..

[20] Jones D.C., Kuhar M.J. (2008). CART receptor binding in primary cell cultures of the rat nucleus accumbens. Synapse.

[21] Somalwar A.R., Ghoudhary A.G., Sharma P.R., Nagalakshmi B., Sagarkar S., Sakharkar A.J., Subhedar N.K., Kokare D.M. (2018). Cocaine- and amphetamine-regulated transcript peptide (CART) induced reward behavior is mediated via Gi/o dependent phosphorylation of PKA/ERK/CREB pathway. Behav. Brain Res..

[22] Hunter R.G., Jones D., Vicentic A., Hue G., Rye D., Kuhar M.J. (2006). Regulation of CART mRNA in the rat nucleus accumbens via D3 dopamine receptors. Neuropharmacology.

[23] Jones D.C., Kuhar M.J. (2006). Cocaine-amphetamine-regulated transcript expression in the rat nucleus accumbens is regulated by adenylyl cyclase and the cyclic adenosine 5′-monophosphate/protein kinase a second messenger system. J. Pharmacol. Exp. Ther..

[24] Rogge G.A., Jones D.C., Green T., Nestler E., Kuhar M.J. (2009). Regulation of CART peptide expression by CREB in the rat nucleus accumbens in vivo. Brain Res..

[25] Beaulieu J.M., Gainetdinov R.R., Caron M.G. (2007). The Akt-GSK3 signaling cascade in the actions of dopamine. Trends Pharmacol. Sci..

[26] Beurel E., Grieco S.F., Jope R.S. (2015). Glycogen synthase kinase-3 (GSK-3): Regulation, actions, and diseases. Pharmacol. Ther..

[27] Barr J.L., Unterwald E.M. (2020). Glycogen synthase kinase-3 signaling in cellular and behavioral responses to psychostimulant drugs. Biochim. Biophys. Acta-Mol. Cell Res..

[28] Perrine S.A., Miller J.S., Unterwald E.M. (2008). Cocaine regulates protein kinase B and glycogen synthase kinase-3 activity in selective regions of rat brain. J. Neurochem..

[29] Miller J.S., Barr J.L., Harper L.J., Poole R.L., Gould T.J., Unterwald E.M. (2014). The GSK3 signaling pathway is activated by cocaine and is critical for cocaine conditioned reward in mice. PLoS ONE.

[30] Kim W.Y., Jang J.K., Lee J.W., Jang H., Kim J.H. (2013). Decrease of GSK3β phosphorylation in the rat nucleus accumbens core enhances cocaine-induced hyper-locomotor activity. J. Neurochem..

[31] Enman N.M., Unterwald E.M. (2012). Inhibition of GSK3 attenuates amphetamine-induced hyperactivity and sensitization in the mouse. Behav. Brain Res..

[32] Ferrario C.R., Li X., Wang X., Reimers J.M., Uejima J.L., Wolf M.E. (2010). The role of glutamate receptor redistribution in locomotor sensitization to cocaine. Neuropsychopharmacology.

[33] Bell K., Kalivas P.W. (1996). Context-specific cross-sensitization between systemic cocaine and intra-accumbens AMPA infusion in the rat. Psychopharmacology.

[34] Pierce R.C., Bell K., Duffy P., Kalivas P.W. (1996). Repeated cocaine augments excitatory amino acid transmission in the nucleus accumbens only in rats having developed behavioral sensitization. J. Neurosci..

[35] Ghasemzadeh M.B., Permenter L.K., Lake R., Worley P.F., Kalivas P.W. (2003). Homer1 proteins and AMPA receptors modulate cocaine-induced behavioural plasticity. Eur. J. Neurosci..

[36] Peineau S., Taghibiglou C., Bradley C., Wong T.P., Liu L., Lu J., Lo E., Wu D., Saule E., Bouschet T. (2007). LTP inhibits LTD in the hippocampus via regulation of GSK3beta. Neuron.

[37] Wang M.J., Li Y.C., Snyder M.A., Wang H., Li F., Gao W.J. (2013). Group II metabotropic glutamate receptor agonist LY379268 regulates AMPA receptor trafficking in prefrontal cortical neurons. PLoS ONE.

[38] Li Y.Z., Tang X.H., Wang C.Y., Hu N., Xie K.L., Wang H.Y., Yu Y.H., Wang G.L. (2014). Glycogen synthase kinase-3beta inhibition prevents remifentanil-induced postoperative hyperalgesia via regulating the expression and function of AMPA receptors. Anesth. Analg..

[39] Nelson C.D., Kim M.J., Hsin H., Chen Y., Sheng M. (2013). Phosphorylation of threonine-19 of PSD-95 by GSK3 is required for PSD-95 mobilization and long-term depression. J. Neurosci..

[40] Paxinos G., Watson C. (1998). The Rat Brain in Stereotaxic Coordinates.

[41] Beaulieu J.M., Sotnikova T.D., Yao W.D., Kockeritz L., Woodgett J.R., Gainetdinov R.R., Caron M.G. (2004). Lithium antagonizes dopamine-dependent behaviors mediated by an AKT/glycogen synthase kinase 3 signaling cascade. Proc. Natl. Acad. Sci. USA.

[42] Beaulieu J.M., Sotnikova T.D., Marion S., Lefkowitz R.J., Gainetdinov R.R., Caron M.G. (2005). An Akt/-arrestin 2/PP2A signaling complex mediates dopaminergic neurotransmission and behavior. Cell.

[43] Beaulieu J.M., Caron M.G. (2008). Looking at lithium: Molecular moods and complex behavior. Mol. Interv..

[44] Hers I., Vincent E.E., Tavaré J.M. (2011). Akt signalling in health and disease. Cell. Signal..

[45] Dalia A., Uretsky N.J., Wallace L.J. (1998). Dopaminergic agonists administered into the nucleus accumbens: Effects on extracellular glutamate and on locomotor activity. Brain Res..

[46] Reid M.S., Hsu K., Berger S.P. (1997). Cocaine and amphetamine preferentially stimulate glutamate release in the limbic system: Studies on the involvement of dopamine. Synapse.

[47] Kalivas P.W., Duffy P. (1997). Dopamine regulation of extracellular glutamate in the nucleus accumbens. Brain Res..

[48] Mao L.M., Diaz J.A., Fibuch E.E., Wang J.Q. (2013). Regulation of phosphorylation of synaptic and extrasynaptic GluA1 AMPA receptors in the rat forebrain by amphetamine. Eur. J. Pharmacol..

[49] Xue B., Edwards M.C., Mao L.M., Guo M.L., Jin D.Z., Fibuch E.E., Wang J.Q. (2014). Rapid and sustained GluA1 S845 phosphorylation in synaptic and extrasynaptic locations in the rat forebrain following amphetamine administration. Neurochem. Int..

[50] Santos S.D., Carvalho A.L., Mao A.L., Caldeira M.V., Duarte C.B. (2009). Regulation of AMPA receptors and synaptic plasticity. Neuroscience.

[51] Dajani R., Fraser E., Roe S.M., Young N., Good V., Dale T.C., Pearl L.H. (2001). Crystal structure of glycogen synthase kinase 3 beta: Structural basis for phosphate-primed substrate specificity and autoinhibition. Cell.

[52] Frame S., Cohen P., Biondi R.M. (2001). A common phosphate binding site explains the unique substrate specificity of GSK3 and its inactivation by phosphorylation. Mol. Cell.

[53] Zahm D.S. (2000). An integrative neuroanatomical perspective on some subcortical substrates of adaptive responding with emphasis on the nucleus accumbens. Neurosci. Biobehav. Rev..

[54] Cardinal R.N., Parkinson J.A., Hall J., Everitt B.J. (2002). Emotion and motivation: The role of the amygdala, ventral striatum, and prefrontal cortex. Neurosci. Biobehav. Rev..

[55] Meredith G.E., Baldo B.A., Andrezjewski M.E., Kelley A.E. (2008). The structural basis for mapping behavior onto the ventral striatum and its subdivisions. Brain Struct. Funct..

[56] Pascoli V., Cahill E., Bellivierl F., Caboche J., Vanhoutte P. (2014). Extracellular signal-regulated protein kinase 1 and 2 activation by addictive drugs: A signal toward pathological adaptation. Biol. Psychol..

[57] Choi J.M., Ahn M.H., Chae W.J., Jung Y.G., Park J.C., Song H.M., Kim Y.E., Shin J.A., Park C.S., Park J.W. (2006). Intranasal delivery of the cytoplasmic domain of CTLA-4 using a novel protein transduction domain prevents allergic inflammation. Nat. Med..

[58] Pellegrino L.J., Pellegrino A.S., Cushman A.J. (1979). A Stereotaxic Atlas of the Rat Brain.

